# 

**Published:** 1846-08

**Authors:** 


Dr. George Chandler has been appointed Superintendent of the Mass. State Lunatic Asylum, at Worcester, Dr. Woodward having resigned the office. Dr. C. was formerly superintendent of the Insane Asylum, at Concord, New-Hampshire. A committee of the citizens of Worcester has been appointed to procure a marble bust of Dr. Woodward, to be placed in the hospital.

---

Dr. Caleb Green has been appointed "Lecturer on Anatomy and Physiology" in the Cortland Academy one of the oldest and most flourishing institutions of the kind in the State. The practice of teaching these sciences in schools and colleges is gradually increasing in favor, and is evidence of a growing appreciation of their value and importance.

Dr. Green is a young man, and yet "not unknown to fame." His occasional contributions to the medical journals are of the right stamp, and prove him to be a sound thinker and most laborious student. If our horoscope does not deceive us, his star is in the ascendant, and the present "conjunction" will prove a most fortunate one for the Cortland Academy.

☞ The Buffalo Medical Association will hold its stated Monthly Meeting on Tuesday Evening, the 4th inst., at Dr. Hamilton's Office, for election of Officers, &c.

